# A multicenter comparison of MOG-IgG cell-based assays

**DOI:** 10.1212/WNL.0000000000007096

**Published:** 2019-03-12

**Authors:** Patrick J. Waters, Lars Komorowski, Mark Woodhall, Sabine Lederer, Masoud Majed, Jim Fryer, John Mills, Eoin P. Flanagan, Sarosh R. Irani, Amy C. Kunchok, Andrew McKeon, Sean J. Pittock

**Affiliations:** From the Oxford Autoimmune Neurology Group (P.J.W., M.W., S.R.I.), Nuffield Department of Clinical Neurosciences, UK; Institute for Experimental Immunology (L.K., S.L.), Affiliated to Euroimmun AG, Luebeck, Germany; and Departments of Neurology (M.M., E.P.F., A.C.K., A.M., S.J.P.) and Laboratory Medicine and Pathology (J.F., J.M., E.P.F., A.C.K., A.M., S.J.P.), Mayo Clinic, College of Medicine, Rochester, MN.

## Abstract

**Objectives:**

To compares 3 different myelin oligodendrocyte glycoprotein–immunoglobulin G (IgG) cell-based assays (CBAs) from 3 international centers.

**Methods:**

Serum samples from 394 patients were as follows: acute disseminated encephalomyelitis (28), seronegative neuromyelitis optica (27), optic neuritis (21 single, 2 relapsing), and longitudinally extensive (10 single, 3 recurrent). The control samples were from patients with multiple sclerosis (244), hypergammaglobulinemia (42), and other (17). Seropositivity was determined by visual observation on a fluorescence microscope (Euroimmun fixed CBA, Oxford live cell CBA) or flow cytometry (Mayo live cell fluorescence-activated cell sorting assay).

**Results:**

Of 25 samples positive by any methodology, 21 were concordant on all 3 assays, 2 were positive at Oxford and Euroimmun, and 2 were positive only at Oxford. Euroimmun, Mayo, and Oxford results were as follows: clinical specificity 98.1%, 99.6%, and 100%; positive predictive values (PPVs) 82.1%, 95.5%, and 100%; and negative predictive values 79.0%, 78.8%, and 79.8%. Of 5 false-positives, 1 was positive at both Euroimmun and Mayo and 4 were positive at Euroimmun alone.

**Conclusions:**

Overall, a high degree of agreement was observed across 3 different MOG-IgG CBAs. Both live cell-based methodologies had superior PPVs to the fixed cell assays, indicating that positive results in these assays are more reliable indicators of MOG autoimmune spectrum disorders.

There is accumulating evidence that CNS inflammatory demyelinating disorders (IDDs), including forms of neuromyelitis optica (NMO) spectrum disorders, acute disseminated encephalomyelitis (ADEM), optic neuritis (recurrent more than single episode), and transverse myelitis are commonly associated with immunoglobulin G (IgG) targeting aquaporin-4 (AQP4) or myelin oligodendrocyte glycoprotein (MOG).^1–5^ Until their relatively recent discovery, patients with these disorders were commonly misdiagnosed as having multiple sclerosis (MS), yet contemporary findings show that MS, MOG-IgG, and AQP4-IgG–associated IDDs have clinical, radiologic, pathologic, and prognostic differences.^5,6^

MOG-IgG–associated IDDs may have a higher prevalence in children and are often relapsing, commonly manifesting as optic neuritis. Attacks may be associated with accumulating neuronal injury and functional impairment. MOG-IgG may be transient or persistent, and its role as a predictor of relapse remains a focus of ongoing study. While MOG antibody has had a checkered past as a biomarker because of a lack of any specific disease association, contemporary methodologies using cell-based assays (CBAs) now define an autoimmune oligodendroglyopathy with a preferential response to immunosuppressants rather than disease-modifying agents (DMA) commonly used in MS.^4–8^ Early initiation and prolonged administration of such drugs may prevent relapses and reduce disability accrual, although randomized clinical trials have not yet been undertaken. MOG-IgG also provides important prognostic information. Hence, accurate serologic diagnosis is imperative to optimize clinical care.

A recent review article published in 2017 by key opinion leaders in the field stated that “methods for detecting MOG antibodies have improved substantially, with cell based assays (CBAs) being state of the art.”^1^ In this blinded study, 3 different MOG-IgG CBAs from 3 international centers were compared.

## Methods

### Standard protocol approvals, registrations, and patient consents

All patients in our study consented to the use of their medical records for research purposes. The study was approved by the Institutional Review Board of Mayo Clinic, Rochester, MN (No. 08-007846).

Serum samples from 394 patients and controls were tested: 91 patients were classified as having a MOG-IgG–like clinical phenotype and included ADEM (28), AQP4-IgG seronegative NMO (27, fulfilling Wingerchuk diagnostic criteria for NMO, either 1999 or 2006 [excluding antibody status]), optic neuritis (21 single, 2 relapsing), or longitudinally extensive transverse myelitis (10 single, 3 recurrent). The control samples were collected from patients with MS (244, selected from the Olmsted County MS population-based cohort), hypergammaglobulinemia (42), and other (17, encephalitis, glioma, Creutzfeldt-Jakob disease, glaucoma). Sensitivity was calculated as the percentage of positives cases within the MOG-IgG–like clinical phenotype cohort. Specificity was calculated as the percentage of positive cases in the MS cohort and those with other neurologic presentations inconsistent with an MOG-related clinical phenotype. Positive predictive value (PPV) was calculated as the percentage of positive test results in patients with MOG-IgG–like clinical phenotypes of all positive test results and estimates the reliability of a positive test result. In contrast, the negative predictive value is the percentage of negative test results in patients without an MOG-IgG–like clinical phenotype of all negative test results and is an estimate of how reliably a negative test result rules out the disease. This study was approved by the Mayo Clinic Institutional Review Board.

All samples were stored at −80°C at the Mayo Clinic central laboratory. They were divided into aliquots and provided frozen as coded samples to the 3 neuroimmunology laboratories: Mayo Clinic; Oxford, UK; and Euroimmun, Germany. All samples were tested by investigators blinded to the clinical information. Methodologies of the 3 assays are shown in table 1, and staining of cells considered positive and negative by all 3 assays is illustrated in the figure

**Table 1:**
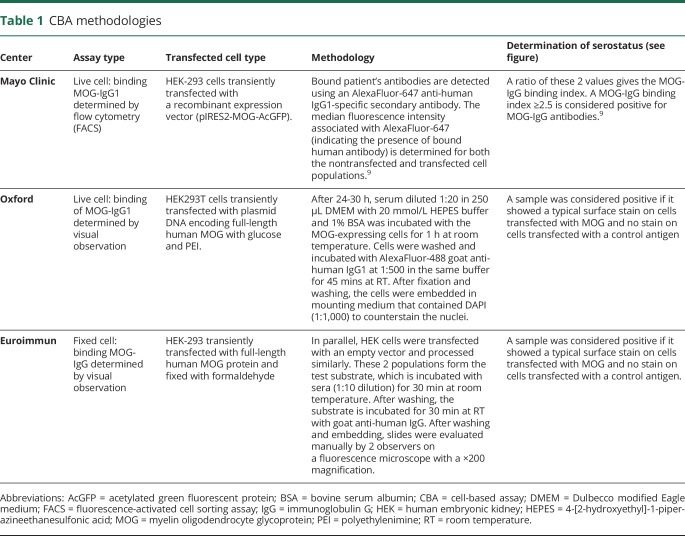
CBA methodologies

**Figure:**
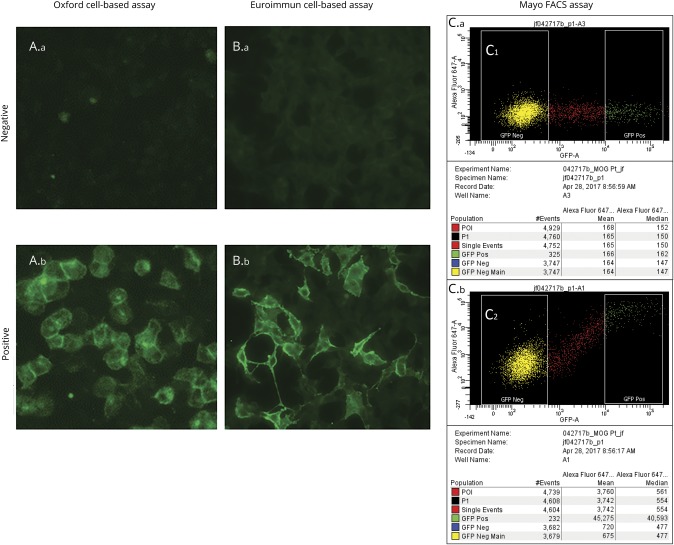
Comparison of positive and negative controls for Oxford, Euroimmun, and Mayo Clinic MOG-IgG assays Oxford cell-based assay (CBA) (A.a) negative and (A.b) positive result; Euroimmun CBA (B.a) negative and (B.b) positive result; and Mayo fluorescence-activated cell sorting assay (FACS) (C.a) negative and (C.b) positive result. For the Mayo FACS assay, 2 cell populations are used to obtain a median fluorescent intensity. The green fluorescent protein (GFP)–negative population represents nontransfected cells, and the GFP-positive population represents cells that express both acetylated GFP and myelin oligodendrocyte glycoprotein (MOG) protein. The AlexaFluor-647 median intensity is an indicator of bound human serum antibodies. As shown in panel (C.b), the positive control has a median AlexaFluor-647 intensity of 40,593 for the GFP-positive population, and the GFP-negative population is 477. The negative control (C.a) has a median 647 intensity of 162 for the GFP-positive population, and the GFP-negative population is similar at 147. These statistical values are used to calculate the immunoglobulin G (IgG) binding index, which is a ratio of the GFP-positive value over the GFP-negative value.

### Data availability statement

The dataset used and analyzed during the current study is available from the corresponding author on reasonable request.

## Results

Of the 25 case samples positive by any methodology, 21 were concordant on all 3 assays, 2 were positive by the Oxford assay and Euroimmun assays, and 2 were positive only by the Oxford assay.

Clinical specificity, as measured using a cohort of 244 patients with MS and 17 patients with disorders clearly outside of the autoimmune MOG spectrum, was 98.1% for Euroimmun, 99.6% for Mayo, and 100% for Oxford. The corresponding PPVs were 82.1%, 95.5%, and 100%, respectively. Negative predictive values were 79.0%, 78.8%, and 79.8%. Of the 5 false-positive findings in this cohort, 1 was positive by both the Euroimmun and Mayo assays (table 2). The additional 4 false-positive results were limited to the Euroimmun CBA. Analytical specificity was high for all 3 assays; no false-positives were identified in a cohort of 42 patients with hypergammaglobulinemia. The results of this multicenter method comparison study of MOG-IgG testing are summarized in table 2. All pairwise comparisons revealed good interassay reliability with κ values >0.8 indicating a high degree of agreement across methods (Cohen κ statistic). Therefore, despite different methodologies and testing locations, the majority of samples achieved the same results across platforms. This is critical for the initial deployment of MOG-IgG–based assays because it provides confidence in the reliability of a positive result but also indicates that detection of MOG-IgG antibodies is robust and that these assays are inherently well standardized.

**Table 2:**
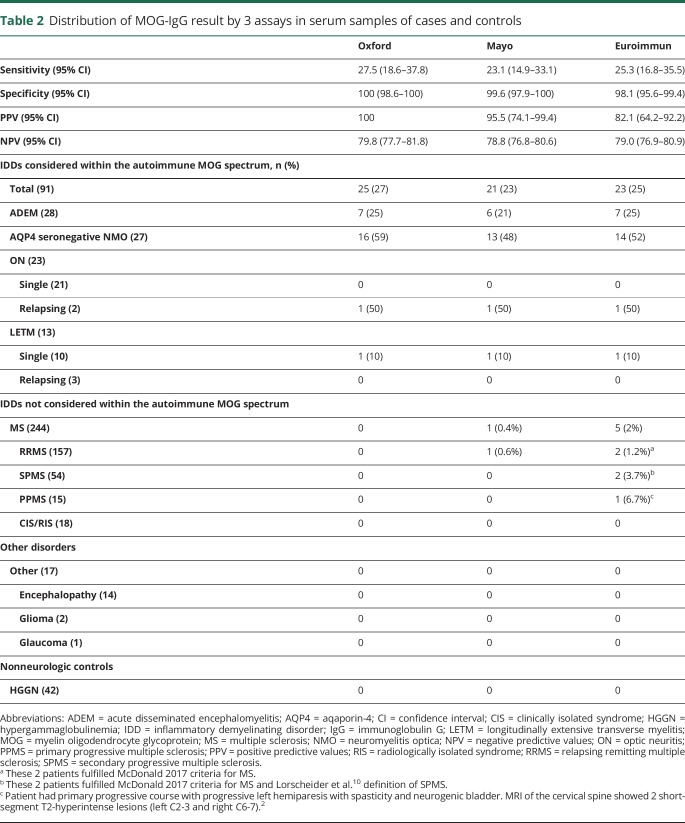
Distribution of MOG-IgG result by 3 assays in serum samples of cases and controls

## Discussion

Both live cell–based methodologies, distinct assays performed at different centers, had superior PPVs to the fixed assays, indicating that positive results in these assays are more reliable indicators of MOG spectrum disorders. ELISA is not a reliable methodology for MOG-IgG detection. MOG-IgG–related diseases may benefit from early and ongoing immunotherapies. Often, inflammatory idiopathic CNS disease such as ADEM, optic neuritis, and transverse myelitis are treated similarly to those with glial antibodies in the acute setting (steroids or plasmapheresis). However, for maintenance immunotherapy, patients without a glial antibody may be less likely to be treated with longer-term immunosuppressants, and longer treatment regimens are associated with fewer relapses in MOG-IgG–related diseases. A false-negative result would often result in a misdiagnosis of MS and consequent treatment with DMAs, which have been reported to worsen AQP4-IgG–positive IDDs, although some are effective for both disorders (anti-CD20 treatments). Data on DMAs exacerbating MOG-IgG disease are currently lacking.

Another concerning consequence of diagnostic inaccuracies is the detection of a false-positive result. Because MOG-IgGs will likely be commonly ordered in the clinical evaluation of a suspected demyelinating event, a false-positive result in a patient with a clinical diagnosis of MS might result in the selection of an immunosuppressant drug (e.g., mycophenolate mofetil, cellcept) rather than a Food and Drug Administration–approved DMA. In this study, 5 of 27 (18.5%) positive results in the commercial test were in control samples, giving a relatively poor PPV (82.1% vs 95.5%–100%). The test is simpler to run in routine diagnostic laboratories, but it has to be fixed to allow transport and storage. The fixation may generate cryptic epitopes, which could explain the clearly positive binding. These discrepancies have also been described in AQP4 assay comparisons. Future studies should address this issue in their design, which may help with a better understanding of this kind of discrepancy.

## Glossary

ADEM: acute disseminated encephalomyelitis
AQP4: aquaporin-4
CBA: cell-based assay
DMA: disease-modifying agent
IDD: inflammatory demyelinating disorder
IgG: immunoglobulin G
IIF: indirect immunofluorescence
MOG: myelin oligodendrocyte glycoprotein
MS: multiple sclerosis
NMO: neuromyelitis optica
PPV: positive predictive value
